# Posterior Interosseous Nerve Compression

**Published:** 2014-01-31

**Authors:** Jeon Cha, Blair York, John Tawfik

**Affiliations:** The Sydney Hospital Hand Unit, Sydney Hospital and Sydney Eye Hospital, Sydney, Australia

**Keywords:** nerve compression, posterior interosseous nerve, radial nerve, radial tunnel, superficial radial nerve

## DESCRIPTION

**Figure 1:**
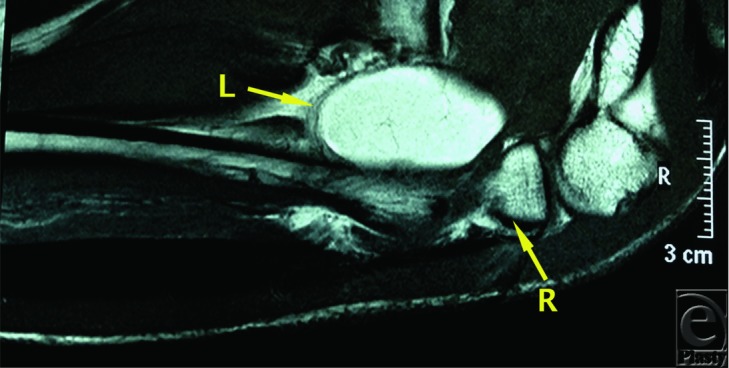
T2 turbo spin-echo weighted magnetic resonance imaging demonstrating a space-occupying lesion in the radial tunnel. L indicates lesion; R, proximal radius.

A 40-year-old man presented with slowly progressing sensory and motor changes to his right upper limb. Examination revealed a mass in his proximal forearm and clinical features that were consistent with radial nerve compression at this level. Magnetic resonance imaging revealed a large mass within the radial tunnel consistent with a lipoma (Fig 1).

## QUESTIONS

1. **What is the anatomy of the radial tunnel?**
2. **What is the course of the posterior interosseous nerve?**
3. **What are the causes of compression of the posterior interosseous nerve?**
4. **What are differences between radial tunnel syndrome and posterior interosseous nerve compression?**

## DISCUSSION

The radial tunnel is a potential space that extends 5 cm from the radial head to the distal margin of supinator. It is bound laterally by the mobile wad (brachioradialis, extensor carpi radialis longus, extensor carpi radialis brevis) and medially by the biceps tendon and brachialis. The roof is formed by the radial recurrent vessels, the superficial head of supinator and brachioradialis. The floor is formed by the capsule of the radiocapitellar joint and the deep head of supinator more distally.^2^^-^6

**Figure 2:**
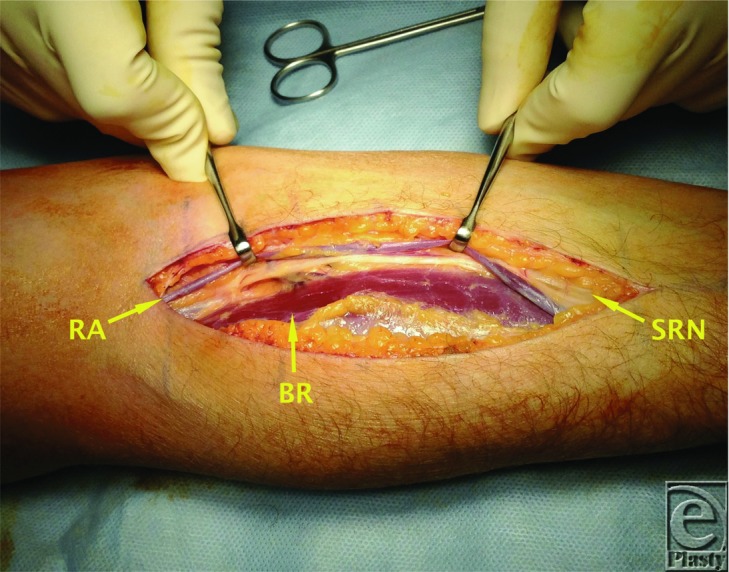
Anterior approach to posterior interosseous nerve decompression. Superficial dissection. BR indicates brachioradialis; RA, radial artery; SRN, superficial radial nerve.

At the elbow, the radial nerve courses beneath the extensor carpi radialis longus and extensor carpi radialis brevis. Approximately 1 to 3 cm distal to the lateral epicondyle it divides into its 2 terminal branches—the superficial radial nerve and the posterior interosseous nerve. The superficial radial nerve is given off proximal to the radial tunnel (Figs 2 and 4) while the posterior interosseous nerve travels within it lying in the fatty tissue anterior to the radiocapitellar joint. The posterior interosseous nerve continues through the radial tunnel, passing beneath the supinator arch to then pierce the deep head of supinator to enter the dorsal compartment of the forearm. Here it divides into a deep and superficial branch and continues distally between the superficial and deep extensor musculature. The posterior interosseous nerve is predominantly a motor nerve and sequentially innervates supinator, extensor carpi radialis brevis, extensor digitorum communis, extensor digiti minimi, extensor carpi ulnaris, abductor pollicis, extensor pollicis brevis, extensor pollicis longus, and extensor indicis. The nerve terminates in the fourth dorsal compartment to give sensory branches to the wrist.^2^^-^6

**Figure 3:**
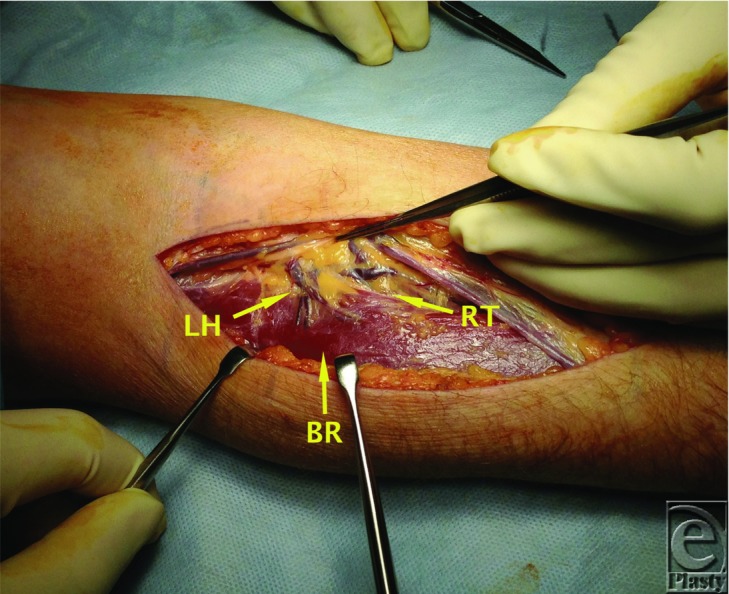
Radial recurrent vessels forming part of the roof of the radial tunnel. BR indicates brachioradialis; LH, Leash of Henry; RT, radial tunnel.

**Figure 4:**
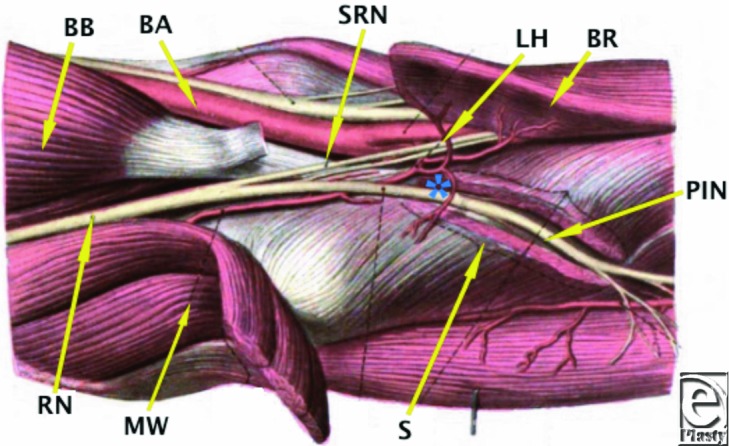
Diagrammatic representation of the branches of the radial nerve as it courses distal to the elbow. Adapted from Ref 1. * indicates level of Arcade of Frohse; BA, brachial artery; BB, biceps brachii; BR, brachioradialis; LH, Leash of Henry; MW, mobile wad; PIN, posterior interosseous nerve; RN, radial nerve; S, deep head of supinator; SRN, superficial radial nerve.

Distal to its origin the posterior interosseous nerve is susceptible to compression at several levels. The first is as it passes the level of the radial head to travel beneath the fibrous bands that are confluent with brachialis, brachioradialis, extensor carpi radialis brevis, and the superficial head of supinator. The second is at the level of the radial neck where the posterior interosseous nerve is crossed by radial recurrent vessels (Leash of Henry) (Figs 3 and 4). The next potential point of compression is beneath the tendinous margin of extensor carpi radialis brevis. The fourth and most common point of compression is as the nerve passes beneath the free aponeurotic margin of supinator (Arcade of Frohse).^7^^,^^8^ The Arcade of Frohse is a connection between the deep and superficial heads of supinator and is fibrotendinous in 30% of the population.^8^ The final potential site of compression is over the distal edge of supinator.^2^^-^6

Radial tunnel syndrome and posterior interosseous nerve entrapment are often used interchangeably as both have the same compressive points. They should, however, be considered as separate entities. Radial tunnel syndrome is associated with pain in lateral aspect of the forearm with tenderness on palpation over the radial tunnel and must be differentiated from lateral epicondylitis. Pain can be exacerbated following activities involving forceful forearm rotation or elbow extension. Provocative maneuvers include resisted forearm supination (with the elbow extended) and resisted middle finger extension. Motor dysfunction is not a feature in radial tunnel syndrome. In contrast, posterior interosseous nerve entrapment is always associated with motor weakness. There may be a history of episodic forearm pain followed by progressive weakness of the extensors of the digits as well as extensor carpi radialis brevis. Symptoms may occur due to trauma (penetrating, tendon rupture, iatrogenic), space-occupying lesions, and inflammatory conditions (rheumatoid arthritis, mononeuritis).^2^ Provocative maneuvers include resisted middle finger extension and supinator compression at the arcade of Frohse during resisted supination.^2^^-^6

The patient in question was found to have a large lipoma within the radial tunnel that had caused pressure atrophy of the supinator and compression of the posterior interosseous nerve. Following its removal, the patients' symptoms improved over a 3-month period. The diagnosis of posterior interosseous nerve compression requires a thorough understanding of the anatomy and the potential clinical causes of the entity.
